# Musculoskeletal Factors and Geriatric Syndromes Related to the Absence of Musculoskeletal Degenerative Disease in Elderly People Aged over 70 Years

**DOI:** 10.1155/2019/7097652

**Published:** 2019-11-18

**Authors:** Shiro Imagama, Kei Ando, Kazuyoshi Kobayashi, Taisuke Seki, Shinya Ishizuka, Masaaki Machino, Satoshi Tanaka, Masayoshi Morozumi, Shunsuke Kanbara, Sadayuki Ito, Taro Inoue, Hiroaki Nakashima, Naoki Ishiguro, Yukiharu Hasegawa

**Affiliations:** ^1^Department of Orthopaedic Surgery, Nagoya University Graduate School of Medicine, 65 Tsurumai, Showa-ku, Nagoya, Aichi 466-8550, Japan; ^2^Department of Rehabilitation, Kansai University of Welfare Sciences, 3-11-1 Asahigaoka, Kashiwara, Osaka 582-0026, Japan

## Abstract

**Purpose:**

To investigate factors with a significant relationship with the absence of musculoskeletal disease (MSD: osteoporosis, knee osteoarthritis (K-OA), and lumbar spondylosis (L-OA)) in elderly people ≥70 years old.

**Methods:**

The subjects were 279 people (134 males, 145 females, mean age: 75.2 years) who attended an annual health checkup and were prospectively included in the study. Osteoporosis was defined as %YAM ≤70%, K-OA as Kellgren–Lawrence grade ≥2, and L-OA as osteophytes of Nathan class ≥3. Subjects were divided into those with (group D) and without (group N) any MSD. Clinical variables including locomotive syndrome (LS), frailty, sarcopenia, and QOL (SF-36) were compared between the groups.

**Results:**

There was no significant difference in age or gender between group N (*n* = 54) and group D (*n* = 225). Lower BMI and pain, including neuropathic pain; greater back muscle strength, physical ability, and balance with eyes closed; larger lumbar lordosis, sacral inclination, and lumbar ROM; and smaller spinal inclination were found in group N. The rates of LS and sarcopenia were significantly lower and QOL was significantly higher in group N.

**Conclusions:**

This study firstly revealed the significant musculoskeletal factors and geriatric syndromes related to an absence of MSD, which may form the basis of interventions to improve QOL in elderly people ≥70 years old.

## 1. Introduction

Musculoskeletal degenerative disease (MSD) increases with age and is common in elderly people, which affect morbidity, quality of life (QOL) and mortality [1]. The three major MSDs are osteoporosis, knee osteoarthritis (K-OA), and lumbar spondylosis (L-OA), and prevention of these MSDs may contribute to an increased QOL because we previously demonstrated that increased comorbidity rates of these MSDs negatively impacted on physical QOL [2]. Obesity may be a cause of K-OA, and this condition has an impact on the spine (knee-spine syndrome) [3–5]. MSDs are also associated with low back pain (LBP), neuropathic pain (NeP), and knee joint pain, and have relationship with muscle strength because muscle exercise can relieve knee joint pain due to K-OA or LBP due to L-OA. To the best of our knowledge, factors related to the absence of MSD in relatively healthy elderly people have not been examined. If these factors can be identified, interventions can be planned to improve QOL in elderly people. Therefore, the objectives of this study were to investigate the rate of absence of MSD and identify factors that are significantly related to an absence of MSD in elderly people ≥70 years old in a health checkup.

## 2. Materials and Methods

A prospective cohort study was conducted at an annual health checkup in Yakumo, Hokkaido, as part of the Yakumo study [6–8]. The checkup has been held annually and supported by local government for over 30 years. A total of 279 subjects aged ≥70 years (male: 134, female: 145, mean age: 75.2 years) were included in the current study. Subjects with a surgical history of osteoporotic fractures, K-OA, and L-OA, or with fresh vertebral fracture were excluded from the study. Osteoporosis was defined as %YAM ≤70% in the calcaneus [9], and K-OA as Kellgren–Lawrence grade ≥2 on plain radiographs of the knee joint [10]. In lumbar plain radiographs, L-OA was defined as osteophytes of Nathan class ≥3 for L1/2-L5/S1 [11]. Spinal sagittal alignment was also examined on these radiographs [12], based on the thoracic kyphosis angle, lumbar lordosis angle, sacral inclination angle, spinal inclination angle, thoracic range of motion (ROM), and lumbar ROM. Measurements were also made using SpinalMouse® (Idiag, Volketswil, Switzerland), which showed high reproducibility compared to plain radiographs without the need for radiation invasiveness in our previous cohort study [13, 14]. A positive lumbar lordosis angle indicates lordosis in this study, and a positive spinal inclination angle indicates a bent forward posture.

Subjects with an absence of MSD were defined as those with no osteoporosis, K-OA, and L-OA (group N). These subjects were compared with those with any MSDs (group D). Age, gender, body mass index (BMI), pain, muscle strength, gait ability, balance, and common geriatric syndromes were examined in each group, Severities of LBP, sciatica, and knee joint pain were evaluated using a visual analogue scale (VAS); 0–100 mm), and neuropathic pain (NeP) was defined as ≥13 points on the pain DETECT questionnaire [15, 16]. QOL was evaluated using the physical component summary (PCS) and the mental component summary (MCS) on SF-36 (Japanese v.2.0) [17, 18].

The study was approved by the Committee on Ethics on Human Research of our University and informed consent was obtained from all subjects.

### 2.1. Muscle Strength Measurement

Grip strength in a standing position was measured once for each hand with a handgrip dynamometer (Toei Light Co., Ltd., Saitama, Japan), and the average value was used [19]. Back muscle strength as the maximal isometric strength of the trunk muscles in a standing posture with 30° lumbar flexion was measured once using a digital back muscle strength meter (T.K.K.5402, Takei Co., Japan) [20].

### 2.2. Physical Ability

Subjects walked a straight 10-m course once at their fastest pace and the time required to complete the course was recorded as the 10 m gait time [21]. In the 3 m timed-up-and-go test (3 m TUG), the time required to rise from a standard chair (46 cm seat height from the ground), walk a distance of 3 m, turn around, walk back to the chair, and sit down was measured. The mean of two trials was recorded. The maximum stride length was measured in a standing position, with subjects asked to put their right foot forward as far as they could, and then to bring the left foot up to the right foot without touching the floor with their hands or knees. This was repeated with the left foot forward, and the average value divided by the subject's height was used as the maximum stride length. In the two-step test, subjects stood with their toes behind a starting line, and took two steps that were as long as possible, and then aligned both feet. The two-step test score was obtained as the length of the two steps (cm) divided by height (cm) [22].

### 2.3. Platform Measurement of Balance

Body balance was assessed with a G-620 stable force platform (Anima, Tokyo, Japan), as previously described [23, 24]. Subjects stood on the foot plate without their shoes and with their arms at their sides and feet close together. The examination was performed twice, each lasting 30 s with eyes open or closed. The distance of movement of the COP per second (LNG/TIME), and the envelopment area traced by movement of the COP (E AREA) were analyzed as measures of balance.

### 2.4. Common Geriatric Syndromes (Locomotive Syndrome, Frailty, and Sarcopenia)

Locomotive syndrome (LS) was proposed by the Japanese Orthopaedic Association (JOA) to define the condition of patients with MSD who may have poor ambulatory status and are at high risk of requiring nursing care [25–27]. The GLFS-25 (score range: 0–100) is a self-administered, but relatively comprehensive, questionnaire of 25 items that are each graded on a 5-point scale from no (0 points) to severe (4 points) impairment, with a higher number indicating greater severity of LS [28]. GLFS-25 scores of ≥16 and ≤15 define subjects with and without LS, respectively [29].

Frailty was first proposed as a disease by Fried et al. [30]. In this study, we used the modified criteria for frailty defined in the Japanese version of the Cardiovascular Health Study [J-CHS] [31]. Frailty was diagnosed when the subjects had ≥3 of 5 criteria: unintentional weight loss (>2 kg in the past 6 months without any particular cause), weakness (decrease of grip strength based on Asian Working Group for Sarcopenia [AWGS] criteria [32], grip strength <26 kg in males and <18 kg in females), low walking speed (usual gait speed <1.0 m/s), self-reported exhaustion, and self-reported low physical activity.

For diagnosis of sarcopenia, appendicular skeletal muscle mass was measured using bioelectrical impedance analysis (BIA) (Inbody 720; Biospace Co., Ltd., Seoul, Republic of Korea) [33]. The BIA reference values for diagnosis of muscle loss are an appendicular skeletal muscle index of <7.0 kg/m^2^ in men and <5.8 kg/m^2^ in women [34, 35]. Sarcopenia in the healthy volunteers in this study was simply defined as a decrease in muscle mass, without inclusion of gait speed or grip strength in the definition.

### 2.5. Statistical Analysis

An unpaired* t* test, Mann–Whitney *U* test, chi-squared test, and multivariate logistic regression analysis with an odds ratio (OR) were used for statistical analysis with SPSS ver.22 (SPSS Inc., Chicago, IL, USA). *p* < 0.05 was considered to be significant in all analyses.

## 3. Results

**Figure 1:**
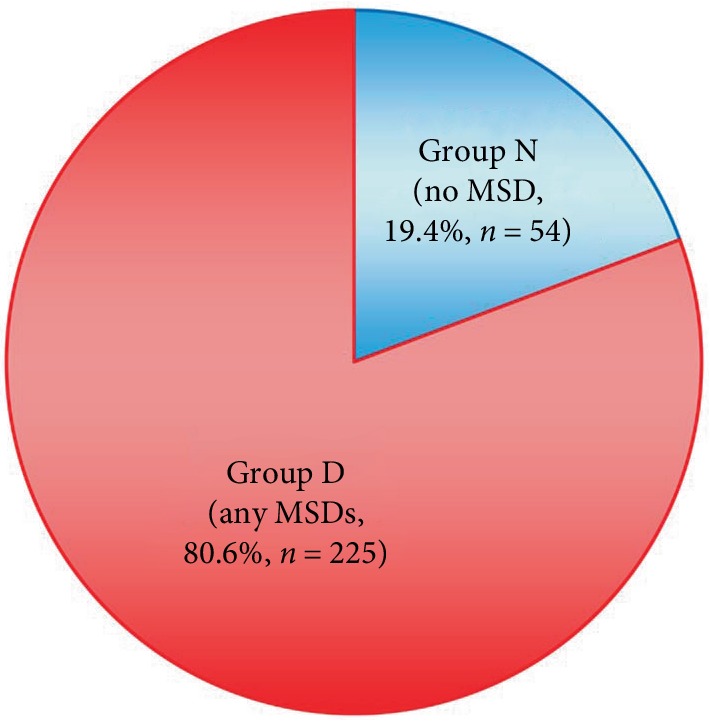
Rate of absence of MSD (group N) in elderly people aged ≥70 years.

The characteristics of the 279 subjects aged ≥70 years are shown in Table 1. Reflecting the nature of Japanese elderly subjects in a health checkup, there were no excessively overweight subjects in this study. The rates of LS, frailty, and sarcopenia were 21.5%, 15.1%, and 25.4%, respectively, and those of osteoporosis, K-OA, and L-OA were 36.2%, 46.6%, and 25.8%, respectively. There were 54 subjects (19.4%) without MSD (group N), and 225 subjects with at least one MSD (group D) (Figure 1). Comparisons between groups N and D are shown in Table 2. Age was similar and gender did not differ significantly between the two groups. The subjects in group N had a significantly lower BMI (*p* < 0.005); significantly higher back muscle strength (*p* < 0.01), maximum stride, and two-step test score (*p* < 0.05); less LBP, sciatica, and knee joint pain; and a lower rate of NeP (*p* < 0.05). Balance with eyes closed was significantly better (*p* < 0.01); the lumbar lordosis angle (*p* < 0.005), sacral inclination angle (*p* < 0.05), and lumbar ROM (*p* < 0.0001) were significantly larger; and the spinal inclination angle was significantly smaller in group N (*p* < 0.05). Thoracic spine alignment did not differ between the groups. The rates of LS (*p* < 0.005) and sarcopenia (*p* < 0.05) were significantly lower in group N, but there was no difference in frailty. Subjects in group N had significantly higher QOL, based on PF, GH, VT, and PCS in SF36 (Table 3, *p* < 0.001).

## 4. Discussion

The absence of MSD was found in 19.4% of a prospective cohort of elderly subjects with a mean age of 75.4 years in this study. The subjects with no MSD had significantly higher QOL in the SF36 physical function, general health, and vitality domains, and the physical component summary, compared to subjects with MSD. Factors associated with an absence of MSD were identified, and these may serve as intervention targets to maintain and improve QOL in elderly people. There was no difference in age or gender between subjects with and without MSD, which permitted identification of BMI, muscle strength, physical ability, pain, balance, spinal parameters, and geriatric syndromes as age- and gender-independent factors associated with an absence of MSD.

High BMI may aggravate mechanical stress on the lumbar spine [36, 37] and joints of the lower extremities [38, 39] in daily life, which suggests that weight control may reduce MSD. In contrast, strong muscles may protect the spine and joints from mechanical stress. We note that back muscle strength, rather than grip strength, was related to absence of MSD, which may be due to inclusion of K-OA and L-OA as MSDs in this study. Back muscle strengthening exercise is used for treatment and prevention of LBP in clinical settings [9, 40], then this back muscle training may reduce L-OA in elderly people, while quadriceps muscle training may be effective for reducing K-OA. Regarding physical ability, two measurements of gait speed were not significantly related to absence of MSD, which may be because solely radiographic degenerative changes do not have a large impact on gait speed in elderly persons over 70 years old. However, the maximum stride and two-step test score were significantly worse in subjects with MSD, which shows that radiographically determined MSD can have a negative impact on physical ability. The severities of LBP, sciatica, and knee joint pain and the NeP rate were lower in subjects without MSD. Severe pain and NeP are clearly a major cause of decreased QOL, as found in previous studies [14, 21, 41]. The results for pain indicate the importance of interventions to reduce MSD in elderly people.

We have previously shown that body balance contributes to prevention of fall [23], and this is important to avoid elderly people becoming bedridden due to fall. We also found that body balance had a significant relationship with spinal inclination, sagittal vertical axis, lumbar lordosis, and sacral inclination [23], which are recently reported the factors for good outcomes in spine surgery [42, 43]. The relationships of the spine with the hips and knees should also be assessed simultaneously in considering global spinal alignment [44], and prevention of osteoporosis, K-OA, and L-OA may maintain spinal sagittal alignment in elderly persons. In this study, thoracic parameters did not differ between subjects with and without MSD, which may be due to exclusion of persons who had undergone spinal surgery or had fresh vertebral fracture. However, thoracic hyperkyphosis is clearly related to osteoporotic vertebral fracture and is a major concern in an aging society, and the thoracic spine should not be neglected in elderly people. Elderly persons should be reminded to maintain a good posture, and to perform muscle exercise to prevent poor spinal sagittal alignment. Stretching and ROM exercises are also important because lumbar ROM was significantly higher in subjects with MSD.

Lastly, regarding geriatric syndromes, there was no significant difference in the frailty rate between the subjects with and without MSD, although this rate showed a tendency to be larger in those with MSD. Therefore, the non-significant result may be due to the small number of cases. LS and sarcopenia differed significantly between the two groups. LS is clearly related to MSD because the JOA proposed LS to define the condition of persons with MSD in high-risk groups. The GLFS-25 questionnaire allows simple screening for LS without other examinations, including radiography, which shows the usefulness of checking LS in elderly people. Sarcopenia in this study was defined as a decrease in muscle mass based on a simple and rapid measurement, and gait ability was not considered. However, sarcopenia in this study was still identified as a significant factor related to an absence of MSD. Dietary changes, supplements, and exercise are required to reduce sarcopenia, and elderly people should be advised to check LS and sarcopenia and recognize conditions of MSD themselves, with the goal of improving their QOL.

There are some limitations in the study. First, the subjects were relatively healthy elderly volunteers who had worked in agriculture or fishing, and this background differs from urban residents. However, there may be particular value in investigating factors related to absence of MSD in these healthy volunteers. Second, the study included only a small number of cases aged ≥70 years. However, the health checkup provides a reliable prospective cohort with complete data that is checked carefully by staff in the Yakumo study. In addition, there was no difference in age or gender between subjects with and without MSD, which permitted identification of BMI, muscle strength, physical ability, pain, balance, spinal parameters, and geriatric syndromes as age- and gender-independent factors associated with an absence of MSD. As the next step, we plan to conduct the multivariate analysis of factors related to the absence of MSD in more subjects with wide range of age. Third, we need a longitudinal intervention study using the key factors found in the current study to determine if these significant factors are a cause or result of absence of MSD.

## 5. Conclusions

The rate of absence of MSD was 19.4% in this study. The significant factors related to absence of MSD were identified as low BMI, greater back muscle strength, good physical ability, mild pain, good body balance, good spinal parameters and ROM, and low rate of geriatric syndrome. This study firstly revealed these significant musculoskeletal factors and geriatric diseases related to an absence of MSD, which may be important intervention targets for improvement of QOL in elderly people aged ≥70 years.

## Figures and Tables

**Table 1:** Characteristics of the 279 subjects aged ≥70 years.

Variable	Value
Age (years)	75.2 (4.5)
Gender (male, %)	48.0% (*n* = 134)
Body mass index (kg/m^2^)	23.5 (3.0)
Grip strength (kg)	26.1 (8.5)
Back muscle strength (kg)	64.7 (27.9)

*Physical ability*	
10 m gait time (s)	5.6 (1.0)
3 m TUG (s)	6.9 (1.3)
Maximum stride (%)	71.1% (7.3)
Two-step test score	1.37 (0.15)

*Pain*	
VAS (low back pain) (mm)	12.0 (19.3)
VAS (sciatica) (mm)	10.7 (19.2)
VAS (knee joint pain) (mm)	14.0 (22.7)
NeP (+) *(n)*	15.4% (*n* = 43)

*Platform measurements of balance*	
LNG/TIME (cm/s)
Eyes open	1.9 (0.77)
Eyes closed	2.5 (1.2)
E AREA (cm^2^)	
Eyes open	2.9 (1.5)
Eyes closed	3.9 (2.7)

*Spinal parameters*
Thoracic kyphosis angle (°)	39.9 (10.2)
Lumbar lordosis angle (°)	12.5 (10.7)
Sacral inclination angle (°)	4.0 (8.3)
Spinal inclination angle (°)	1.8 (4.8)
ROM (thoracic spine)	11.6 (14.8)
ROM (lumbar spine)	42.1 (13.6)

*Geriatric disease*
LS (%) (*n*)	21.5% (*n* = 60)
Frailty (%) (*n*)	15.1% (*n* = 42)
Sarcopenia (%) (*n*)	25.4% (*n* = 71)

*Musculoskeletal degenerative disease*
%YAM	74.4% (11.9)
Osteoporosis (%)	36.2% (*n* = 101)
K-OA (%)	46.6% (*n* = 130)
L-OA (%)	25.8% (*n* = 72)

*QOL*
PCS (SF-36)	45.6 (12.1)
MCS (SF-36)	53.8 (7.6)

Values are shown as a mean or percentage (SD or number of patients (*n*) in parentheses). TUG: timed up and go test, VAS: visual analogue scale, NeP: neuropathic pain, LNG/TIME: distance of movement of the center of pressure (COP) per second, E AREA: envelopment area traced by movement of the COP, ROM: range of motion, LS: locomotive syndrome, YAM: young adult mean, K-OA: knee osteoarthritis, L-OA: lumbar spondylosis, PCS: physical component summary, MCS: mental component summary. Positive value indicates lordosis of the lumbar spine in this study.

**Table 2:** Comparison of parameters in patients without (group N) and with (group D) MSD.

Variables	Group N (*n* = 54)	Group D (*n* = 225)	*p* value
Age (years)	75.4 (4.0)	75.1 (4.6)	NS	
Gender (male, %)	59.3% (*n* = 32)	45.3% (*n* = 102)	NS
**Body mass index (kg/m^2^)**	**22.5 (2.6)**	**23.7 (3.1)**	**<0.005**
Grip strength (kg)	27.3 (10.2)	25.8 (8.0)	NS
**Back muscle strength (kg)**	**73.8 (28.6)**	**62.6 (27.3)**	**<0.01**
*Physical ability*			
10 m gait time (s)	5.8 (1.3)	5.5 (0.97)	NS
3 m TUG	6.6 (1.5)	6.9 (1.2)	NS
**Maximum stride (%)**	**73.5 (6.3)**	**70.5 (7.4)**	**<0.01**
**Two-step test score**	**1.42 (0.15)**	**1.35 (0.14)**	**<0.005**
*Pain*			
**VAS (low back pain) (mm)**	**7.8 (14.9)**	**13.0 (16.1)**	**<0.05**
**VAS (sciatica) (mm)**	**5.0 (8.2)**	**12.0 (20.7)**	**<0.05**
**VAS (knee joint pain) (mm)**	**7.0 (14.3)**	**15.6 (24.0)**	**<0.05**
**NeP (+) (*n*)**	**5.6% ( ** **n** = 3** )**	**17.8% ( ** **n** = 40** )**	**<0.05**
*Platform measurements of balance*			
**LNG/TIME (cm/s)**			
Eyes open	1.8 (0.54)	2.0 (0.81)	NS
**Eyes closed**	**2.2 (0.69)**	**2.6 (1.3)**	**<0.05**
**E AREA (cm^2^)**			
Eyes open	2.6 (1.2)	2.9 (1.5)	NS
**Eyes closed**	**3.1 (1.3)**	**4.1 (2.9)**	**<0.01**
*Spinal parameters*			
Thoracic kyphosis angle (°)	41.4 (8.1)	39.5 (10.6)	NS
**Lumbar lordosis angle (°)**	**17.1 (6.1)**	**11.5 (11.2)**	**<0.005**
**Sacral inclination angle (°)**	**6.0 (7.5)**	**3.5 (8.4)**	**<0.05**
**Spinal inclination angle (°)**	**0.43 (3.7)**	**2.1 (5.0)**	**<0.05**
ROM (Thoracic spine)	13.2 (11.3)	11.2 (15.5)	NS
**ROM (Lumbar spine)**	**49.4 (12.8)**	**40.4 (13.2)**	**<0.0001**
*Geriatric disease*			
**LS (%)** (**n**)	**7.4% **(**n** = 4**)**	**24.9% ( ** **n** = 56** )**	**<0.005**
Frailty (%) (*n*)	20.4% (*n* = 11)	13.8% (*n* = 72)	NS
**Sarcopenia (%)** (**n**)	**16.7% ( ** **n** = 9** )**	**27.6% ( ** **n** = 62** )**	**<0.05**

Values are shown as a mean or percentage (SD in parentheses).

TUG: timed up and go test, VAS: visual analogue scale, NeP: neuropathic pain, LNG/TIME: distance of movement of the center of pressure (COP) per second, E AREA: envelopment area traced by movement of the COP, ROM: range of motion, LS: locomotive syndrome, NS: not significant.

Positive value indicates lordosis of the lumbar spine in this study.

Bold indicates significance.

**Table 3:** QOL (SF-36) in patients without (group N) and with (group D) MSD.

Variables	Group N	Group D	*p* value
**PF**	**90.6 (12.3)**	**82.2 (18.0)**	**<0.001**
RF	86.1 (19.2)	81.9 (23.0)	NS
BP	74.6 (24.8)	70.0 (21.6)	NS
**GH**	**66.1 (15.3)**	**59.1 (19.0)**	**<0.05**
**VT**	**71.9 (18.1)**	**67.2 (16.9)**	**<0.05**
SF	93.1 (12.1)	89.6 (16.7)	NS
RE	88.9 (17.9)	84.8 (23.4)	NS
MH	80.5 (16.4)	77.7 (16.0)	NS
**PCS**	**48.6 (9.9)**	**44.9 (12.5)**	**<0.05**
MCS	54.9 (7.2)	53.5 (7.7)	NS

QOL: quality of life, PF: physical functioning, RP: role-physical, BP: bodily pain, GH: general health perception, VT: vitality, SF: social functioning, RE: role-emotional, MH: mental health, PCS: physical component summary, MCS: mental component summary, NS: not significant. Bold indicates significance.

## Data Availability

The cohort data used to support the findings of this study are restricted by the Institutional Review Board of Nagoya University Graduate School of Medicine in order to protect the privacy of subjects in Yakumo study.
